# Organic Phase Change Nanoparticles for in-Product Labeling of Agrochemicals

**DOI:** 10.3390/nano5041810

**Published:** 2015-10-28

**Authors:** Miao Wang, Binh Duong, Ming Su

**Affiliations:** 1 Department of Chemical Engineering, Northeastern University, Boston, MA 02115, USA; E-Mail: wang.mia@husky.neu.edu; 2 Department of Chemistry, University of California, Santa Barbara, CA 93106, USA; E-Mail: bduong07@gmail.com

**Keywords:** chemicals, anti-counterfeiting, phase change nanoparticles, in-product barcodes

## Abstract

There is an urgent need to develop in-product covert barcodes for anti-counterfeiting of agrochemicals. This paper reports a new organic nanoparticle-based in-product barcode system, in which a panel of organic phase change nanoparticles is added as a barcode into in a variety of chemicals (herein agrochemicals). The barcode is readout by detecting melting peaks of organic nanoparticles using differential scanning calorimetry. This method has high labeling capacity due to small sizes of nanoparticles, sharp melting peaks, and large scan range of thermal analysis. The in-product barcode can be effectively used to protect agrochemical products from being counterfeited due to its large coding capacity, technical readiness, covertness, and robustness.

## 1. Introduction

Counterfeited agrochemicals are often made with low quality reactants, and contain high level of unknown or banned chemicals, which can pollute the environment and cause health issue for farmers, and damage crops. Such products can contaminate ground water or soil, and damage beneficial insects and other organisms [1,2]. Counterfeited agrochemicals threaten agriculture’s ability to provide safe healthy food in sufficient quantities to the society. Unknown and untested residue can enter the food chain and damage customers. Counterfeited agrochemicals cause significant economic damages. The use of counterfeited agrochemicals has caused an approximate 4 billion dollar lost revenue worldwide, and uncountable lost in food production and human safety issues. The European Crop Protection Association estimates that 5%–9% of the market consists of illegal pesticides, often due to counterfeit products with fake labels and packaging.

Barcodes have been used ubiquitously to tag products including agrochemical products, but common barcodes that are attached on packing can be altered or duplicated, facing increasing challenge from counterfeiting and unlawful uses [3,4]. There is an unmet need to develop covert or invisible barcodes that can be added into products for anti-counterfeiting purposes, but existing techniques are inappropriate for covert operation due to large size, visibility, low capacity, and possibility of losing code integrity [5] Radio frequency identifier (RFID) is expensive, cannot be mixed with products and cannot function without structural integrity [6,7]. Chemical and fluorescent taggants are low-cost and easy-to-read, but have small code space, low sensitivity and secureness and are only suited for simple authentication rather than serialization application; even under such condition, they are subject to be counterfeited due to ubiquitous nature [8,9,10]. Glass or plastic microbeads and microfibers have been added in objects as taggants. The morphology and length of microbead/fibers can be analyzed with optical microscope to determine origin (*i.e.*, country or producers) of the product [11,12,13]. Microfibers are embedded in objects such as paper documents, and used as intangible taggants, where fiber morphology can be analyzed for authentication, but it takes a long time to determine morphological character of microfibers. Not only do they have low coding capacity, microfibers and microbeads also lack sensitivity, secureness and covertness needed for the next generation barcodes. It is possible to find relatively large fibers or beads (with diameter of 100 µm) with the naked eye.

Nanomaterials such as nanoparticles, nanowires/tubes can be produced at high yield in controlled manners [14,15,16]. These nanomaterials are invisible to the naked eye, can be directly added into objects of interest without being noticed, and have shown potential as taggants, where the unique properties of nanoparticles including optical, magnetic, electric and electro-chemical ones can be used for readout without signal amplification [17,18,19]. However, the use of nanoparticles to tag each object in a large number of objects is seriously limited due to lack of particle-specific signature, and small coding space. There is no nanoparticle-specific magnetic or electrochemical property, meaning one type of nanoparticles is indistinguishable from others based on magnetic or electrochemical property. Quantum dots or metallic nanoparticles have shown size-dependent optical properties, but the fluorescent and plasmonic emissions have broad peaks with peak width at half height of 150 nm in ultraviolet-visible region, which limits type of optically distinguishable nanoparticle between 400 and 900 nm to only a few [20,21,22]. In the cases that plasmonic metallic nanoparticles are used to enhance sharp Raman scattering peaks over a wide wavelength range, available Raman dyes are limited and quantitative signals are hard to obtain [23]. Metallic nanobarcodes have been produced by electrochemically depositing alternating layers of metals into nanochannels, but the fabrication process takes a long time, and the multi-segment nanobarcodes only provide limited coding ability [24]. In addition, the nanobarcode requires a high resolution optical microscope to detect the optical contrasts of adjacent segments, and is not suitable for rapid readout.

**Figure 1:**
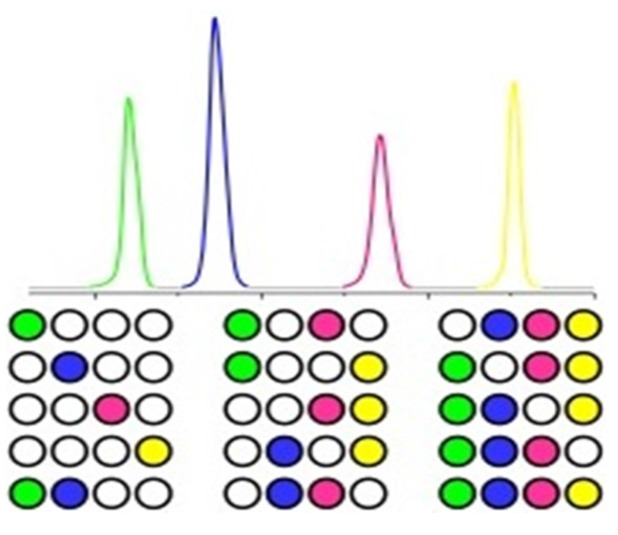
In product barcodes based on phase change nanomaterials for agrochemical products.

This article describes a new nanoparticle based barcode system, where a panel of organic solid-to-liquid phase change nanoparticles is added into an object and used as a thermal barcode to tag agrochemicals (Figure 1). This nano application can avoid modification because nanoparticles are invisible to common users, and only visible to people with advanced technology. The organic nanoparticles will not present any toxic effect to corps, farmers, or the environment [25]. The melting temperature of each type of nanoparticle is derived using differential scanning calorimetry (DSC). Nanoparticles are made with diameter larger than the critical diameter (20 nm) to avoid melting temperature change [26]. Pure substances and eutectic mixtures of organic solids are designed and used to make nanoparticles. The eutectic mixtures go directly from solid phase to liquid phase without pasty stage, and will have sharp melting peaks as those of pure substances [27]. If 10 organic solids can be found such that any two of them form binary eutectic mixtures, the total number of pure organic solids and eutectic mixtures will reach 1023:

$$\overset{n}{\underset{k=1}{\sum }}C_{n}^{k}-1\approx \overset{n}{\underset{k=1}{\sum }}\frac{n!}{k!\left(n-k\right)!}-1=2^{n}-1$$

where *n* is the total number of solids, and k is the number of solids in a nanoparticle [27].

## 2. Results and Discussion

**Figure 2:**
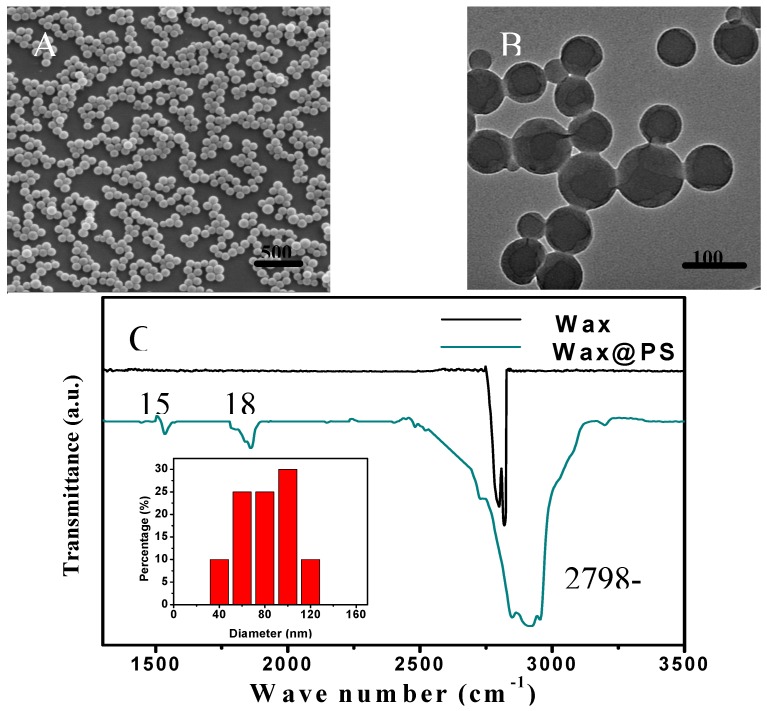
(**A**) Scanning electron microscope (SEM) and (**B**) transmission electron microscope (TEM) images of polymer encapsulated nanoparticles. (**C**) Fourier Transform infrared spectrometer (FTIR) spectra of paraffin (1) and polymer-encapsulated paraffin nanoparticles (2); size distribution of polymer encapsulated nanoparticles (C inset).

Polymer-encapsulated paraffin nanoparticles are characterized as follows. SEM image shows well dispersed spherical nanoparticles (Figure 2A). The fact that there is no nanoparticle aggregation means the nanoparticles can be dispersed evenly in solid powder. The diameters of most nanoparticles are in the range of 90–120 nm, and the average diameter of nanoparticles is 100 nm. TEM image (Figure 2B) clearly shows the core-shell structure of polymer encapsulated nanoparticles. The weak contrast between polymer shell and nanoparticle core is likely due to the fact that both have similar electronic density. While the reason that the core shell structure can be discerned is due to the fact that there is a thin layer of vacuum or void between the core and shell. The void or vacuum layer is observed, because it interacts with electron differently from organic species. FTIR spectrum confirms the formation of polystyrene shell around paraffin core (Figure 2C). The peaks from 2958 to 2843 cm^−1^ are the characteristic peaks of aliphatic C–H stretching vibration. The peaks at 1537 cm^−1^ are associated with benzene ring C=C stretching vibration; and those at 1857 cm^−1^ are benzene ring C=C out-plane bending vibrations. The control sample (*i.e.*, paraffin wax) only has two peaks at 2798 and 2817 cm^−1^ associated with aliphatic C–H stretching vibration [28].

**Figure 3:**
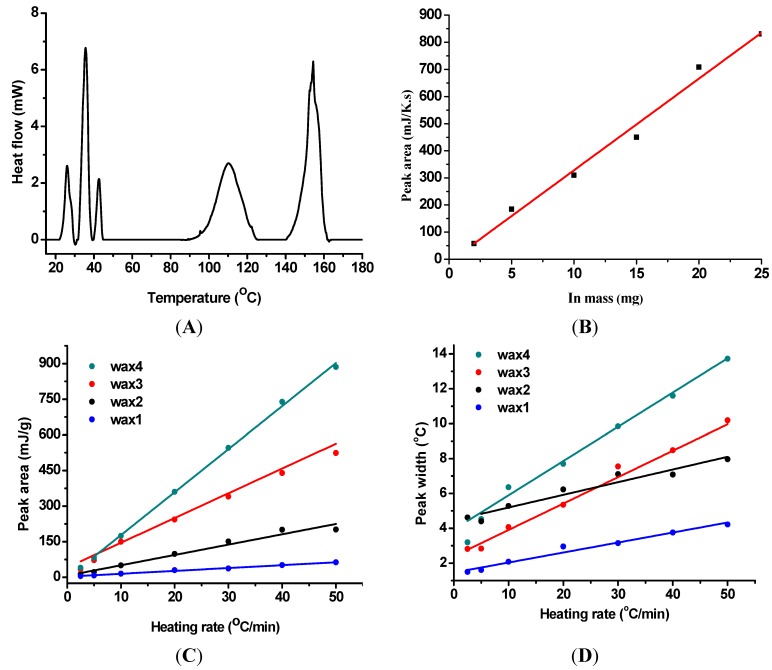
Differential scanning calorimetry (DSC) curve of four paraffin nanoparticles in paclobutrazol (**A**); mass dependent peak area of paraffin nanoparticles (**B**); peak areas (**C**) and peak widths (**D**) of paraffin nanoparticles at different heating rates.

Phase change nanoparticles of paraffin and fatty acids are used to code paclobutrazol (a solid plant food). The nanoparticles are extremely small and can form homogeneous mixture indistinguishable from agrochemical powders which are at micrometer scale. Owing to the large fusion heat of nanoparticles, adding a small amount of these nanoparticles into the agrochemical powders can give strong thermal signal in DSC. In addition, these phase change materials (*i.e.*, paraffin and fatty acids) are not harmful to food and easy to degrade in the environment. As each peak can be taken as a bar, multiple peaks can be treated as a barcode. Figure 3A shows the DSC curve of a barcode (four kinds of paraffin nanoparticles) in paclobutrazol power, where the amount of nanoparticles added is 0.5% of total mass. The peak at 150 °C is from paclobutrazol itself, and those from paraffin nanoparticles are at 28 (wax 1), 37 (wax 2), 46 (wax 3), and 119 °C (wax 4). The peak of wax 4 is wider than others, likely due to the low thermal conductivity of polymer. The nanoparticle barcode has been readout reliably for more than 50 times without losing code information. Increasing the amount of nanoparticles leads to higher detection sensitivity. Figure 3B shows the linear relation between the peak area and the nanoparticle mass, which is in good agreement with the followed Equation:

*Q* = *mC_p_*β

where *Q* is the heat flow (area of melting peak); *m*, *C_p_* and β are mass, specific heat of sample, and heating rate, respectively. Figure 3C shows the linear relation between heating rate and peak area for nanoparticles, where the peak area decreases as heating rate decreases. Therefore, another way to increase detection sensitivity is to run DSC analysis at higher heating rate. The relation between peak width and heating rate (Figure 3D) has two meanings: (1) more peaks can be accommodated in the same barcode at a lower heating rate, which will provide a higher labeling capacity; and (2) the decoding time can be reduced at higher heating rate (at the cost of labeling capacity). The existence of tradeoff and benefit suggests that DSC operations can be tuned to match the need of a particular application [29].

**Figure 4:**
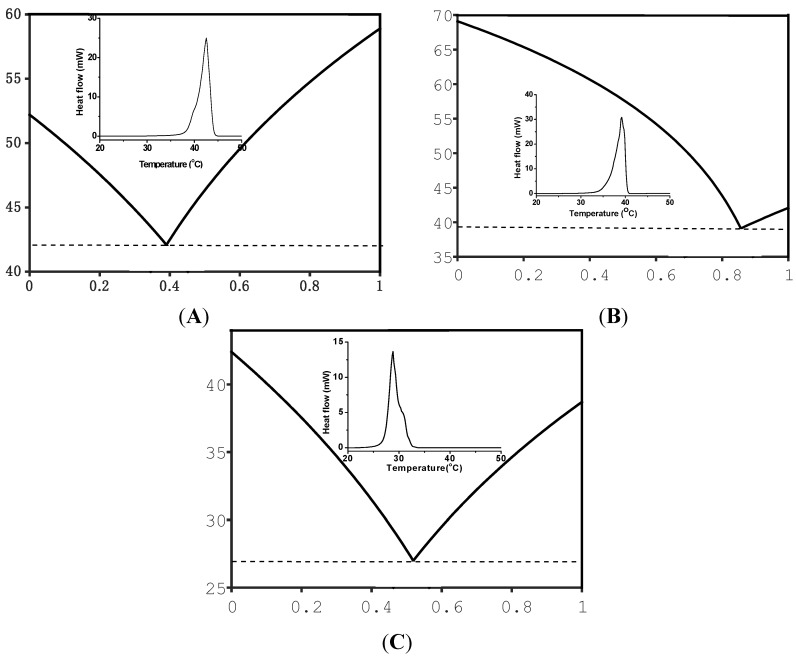
Calculated phase diagram and DSC curves (inset). (**A**) palmitic acid and myristic acid; (**B**) the eutectic mixture of two acids (*i.e.*, palmitic acid and myristic acid) and stearic acid; (**C**) the eutectic mixture of three acids (*i.e.*, palmitic acid-myristic acid-stearic acid) and lauric acid.

Eutectic organic mixtures with defined melting temperature can be form by melting two or more organic compounds such as fatty acids at specific ratio. A MATLAB software is used to derive the eutectic compositions and melting temperatures [30]. The software is built based on calculation of total Gibbs energy as a function of molar ratio of compounds in a system. At any given temperature, pressure, and composition, Gibbs free energy will be at the lowest when all phases reach thermodynamic equilibrium [31]. Figure 4A shows the calculated phase diagram of palmitic acid (PA) and myristic acid (MA), where the melting point and eutectic composition are at 41.6 °C and 39% (PA), respectively. The eutectic composition is then used to design eutectic mixture and make nanoparticles. PA-MA eutectic nanoparticles melt at 42 °C (Figure 4A inset), which is in agreement with the calculated melting temperature. Each eutectic mixture can be taken as a new compound and used to form higher ordered eutectic mixture. Figure 4B shows the phase diagram formed by the binary eutectic mixture (PA-MA), and stearic acid (SA), where the ternary eutectic composition and melting temperature are at 86% and 38.7 °C, respectively. PA-MA-SA eutectic nanoparticles melt at 39 °C (Figure 4B inset), which is in agreement with the calculated melting temperature. Figure 4C showed the phase diagram formed by the ternary eutectic mixture (PA-MA-SA) and lauric acid (LA), where the quaternary eutectic melting peak at 29 °C is close to the calculated one (Figure 4C inset). The difference between the calculated and measured temperatures is likely due to error in calculation [32].

**Figure 5:**
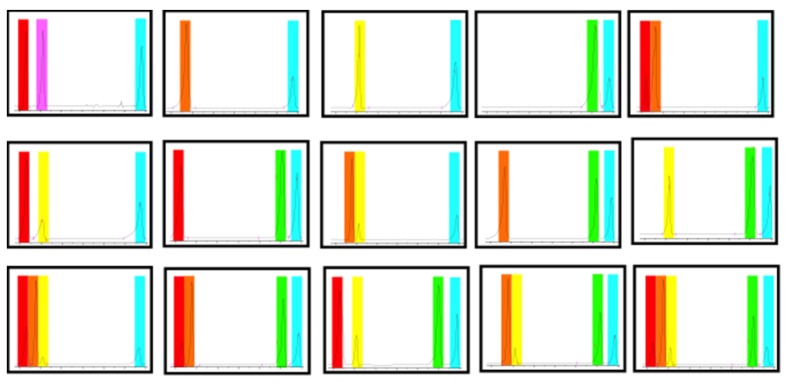
Barcoding agrochemical products with a library of barcodes formed by five types of organic solid nanoparticles.

Nanoparticle barcodes can be formed by adding a mixture of nanoparticles with different mass ratio into objects. Each melting peak can be denoted as one or zero depending on whether heat flux is higher or lower than the detection limit. The information is coded by adding 5 mg of a mixture in an insect killer (solid) at mass ratio of 0.5%, and decoded by putting certain amount of mixture into an aluminum pan and counting the melting peaks in temperature rise process. Figure 5 shows 15 barcodes formed by five types of nanoparticles: octadecane (28 °C), docosane (37 °C), eicosane (46 °C), polywax (119 °C), fatty acid eutectic mixture (42 °C). The melting peak at 136 °C (green colored) is from insect killer itself. There is no chemical or physical reaction due simply to the mixture with insect killer powders under room temperature (20 °C). Each DSC curve is flattened to remove the slope and smoothened to remove thermal fluctuation. The time it takes to readout one code at ramp rate of 10 °C/min is about 5 min, and the decoding time can be reduced by increasing ramp rates.

## 3. Experimental Section

All chemicals used in this experiment are obtained from Aldrich without purification. A one-pot method is used to make polymer-encapsulated nanoparticles of paraffin wax and fatty acids [28]. Briefly, 10.0 g of octadecane at melting point of 28 °C, 1.0 g of acrylate acid and 0.1 g of dodecylmercaptan are dissolved in 10.0 g of styrene by sonication for 10 min. The mixture is poured into 30.0 g of aqueous solution that contains 0.2 g of sodium dodecylsulfate and 0.2 g of Triton X-100 in a 250 mL three-neck flask. The mixture is sonicated for 10 min to form a stable emulsion. The flask is purged by nitrogen for 30 min, followed by heating at 70 °C for 12 h at 200 rpm to complete the polymerization.

The encapsulated phase change nanoparticles have been characterized using a variety of techniques. A differential scanning calorimeter (PerkinElmer DSC7, PerkinElmer, Waltham, MA, USA) is used to measure the thermal properties of nanoparticles at temperature ramp rate of 10 °C/min. A Zeiss (Ultra 55, Carl Zeiss, Jena, Germany) scanning electron microscope (SEM) operated at 10 keV is used to image the morphology of nanoparticles. A transmission electron microscope (TEM, JEOL 1011, Carl Zeiss, Jena, Germany) operated at 100 kV is used to determine the structure of the nanoparticles. In order to prepare sample for TEM imaging, an ethanol drop containing nanoparticles is dropped on a copper TEM grid coated with carbon film. The size distribution of nanoparticles is determined with dynamic light scattering (DLS, PD2000, Precision, Franklin, MA, USA) by adding 5 μL nanoparticle suspension into 1.5 mL water in a plastic cuvette. A Nicolet Avatar 330 Fourier transform infrared spectrometer (FTIR, Vertex 70, Bruker, Ettlingen, Germany) is used to confirm the spectroscopic characters of polymer encapsulated nanoparticles.

## 4. Conclusions

A new in-product barcode system based on a panel of organic solid nanoparticles has been demonstrated for agrochemical products. The nanoparticles have discrete and sharp melting peaks, and have been encapsulated in polymer shells, and mixed with agrochemical products for anti-counterfeiting purpose. The key differentiators of this technique from previous barcodes are: (1) We proved in this article that the nanoparticles with binary, ternary and quaternary compositions can be designed, and synthesized to have melting temperatures close to those predicted with thermodynamic calculation; (2) Covertness. Nanoparticles are invisible to the naked eye and cannot be modified or removed from products; (3) Large coding space. Phase change nanoparticles have large coding space, and can be used to label a large group of objects; (4) High sensitivity. Barcodes can be detected with high sensitivity through thermal readouts; (5) Wide applicability. The in-product barcodes will greatly enhance labeling capacity for commercial and forensic applications by tracking each batch of agrochemical products from producer to customer.
